# Who Stayed Home Under Safer-at-Home? Impacts of COVID-19 on Volume and Patient-Mix at an Emergency Department

**DOI:** 10.5811/westjem.2020.12.49234

**Published:** 2021-02-08

**Authors:** Chun Nok Lam, Sarah Axeen, Sophie Terp, Elizabeth Burner, Daniel A. Dworkis, Sanjay Arora, Michael Menchine

**Affiliations:** * University of Southern California, Keck School of Medicine, Department of Emergency Medicine, Los Angeles, California; † USC Schaeffer Center for Health Policy and Economics, Los Angeles, California

## Abstract

**Introduction:**

To describe the impact of COVID-19 on a large, urban emergency department (ED) in Los Angeles, California, we sought to estimate the effect of the novel coronavirus 2019 (COVID-19) and “safer-at-home” declaration on ED visits, patient demographics, and diagnosis-mix compared to prior years.

**Methods:**

We used descriptive statistics to compare ED volume and rates of admission for patients presenting to the ED between January and early May of 2018, 2019, and 2020.

**Results:**

Immediately after California’s “safer-at-home” declaration, ED utilization dropped by 11,000 visits (37%) compared to the same nine weeks in prior years. The drop affected patients regardless of acuity, demographics, or diagnosis. Reductions were observed in the number of patients reporting symptoms often associated with COVID-19 and all other complaints. After the declaration, higher acuity, older, male, Black, uninsured or non-Medicaid, publicly insured, accounted for a disproportionate share of utilization.

**Conclusion:**

We show an abrupt, discontinuous impact of COVID-19 on ED utilization with a slow return as safer-at-home orders have lifted. It is imperative to determine how this reduction will impact patient outcomes, disease control, and the health of the community in the medium and long terms.

## INTRODUCTION

### Background

In Los Angeles County (LAC) more than 1 million individuals have tested positive for coronavirus disease 2019 (COVID-19) and more than 14,000 individuals have died as of January 20, 2021.^1^ Emergency departments (ED) are at the forefront of the healthcare response to the pandemic, and urban, safety-net public hospitals have been disproportionately impacted by the increase in morbidity and mortality attributed to COVID-19.^2^ With more than 150,000 ED visits annually, the LAC+USC Medical Center is normally one of the busiest EDs in the United States. However, in line with numerous accounts in the popular and academic press, the overall number of patients presenting to the LAC+USC ED plummeted in the wake of “safer-at-home” declarations. Specialties ranging from cardiology^3^–^5^ to emergency medicine^6^ and otolaryngology^7^ noted marked decreases in patient visits for both acute and chronic conditions. This decreased volume allowed providers to focus their efforts on treating COVID-19 patients in a new world of routine personal protective equipment and ”hot zones” within the department without the added stress of facing crowded EDs; however, the patients who avoided the ED may have been placing themselves in danger by skipping needed and emergent medical care.

### Importance

While much of the academic research comes from international settings or focuses on the acute treatment of suspected COVID patients,^8^ accounts in the popular press and commentary pieces in medical literature highlight the danger of patients delaying needed care.^9^–^11^ Emerging academic research from the US bolsters these concerns, showing a 40–60% reduction in ED utilization in the wake of the COVID-19 pandemic and an increase in inpatient admissions as the pandemic intensified.^12^ As these articles point out, the question remains as to what the spillover effects have been and will continue to be of these safer-at-home orders and the COVID-19 pandemic on healthcare utilization and outcomes for patients. In addition to the observation that patients have been reluctant to seek care, there is increasing evidence that the impact of COVID-19 in the US has disproportionately affected populations including Blacks, Latinos, patients with pre-existing health conditions, and lower-income individuals.^13^–^15^ As LAC+USC serves many of these populations, it is key to understand whether the decline in ED utilization has had unequal or disproportionate effects on vulnerable patient populations.

### Goals of This Investigation

The goal of this investigation was to describe the impact of the COVID-19 pandemic on operations of a large, urban, public ED in Los Angeles, California. We characterize the effect of the COVID-19 pandemic and California’s safer-at-home order on ED visits, patient disposition, and diagnosis-mix of ED visits compared to prior years. We show the impact of COVID-19 on changes to patient demographics (age, gender, race/ethnicity), primary payor, and geographic distribution of patients visiting the ED. We describe the differential return of patients to the ED in the weeks following the safer-at-home declarations.

## METHODS

### Study Design and Setting

This investigation is a retrospective analysis of all ED encounters for the first 18 weeks of the year (January to early May) in 2018, 2019, and 2020 at the LAC+USC Medical Center ED. All non-HIV-related ED encounters are included. The analysis focuses on the years 2018, 2019, and 2020 to represent ED volume across two influenza seasons and the COVID-19 pandemic. We selected the first 18 weeks of the calendar year because it includes the peak and downturn of influenza seasons and the implementation of LA County’s safer-at-home order, as well as the predicted or modeled beginning, peak, and downturn of COVID-19 in LA County at the time of the analysis.^16^ The LA County safer-at-home order issued March 19, 2020, shuttered all non-essential businesses, banned all gatherings of more than 10 individuals, and required essential businesses to practice social distancing, provide access to effective hand sanitizer or hand-washing, and to follow any additional communicable disease control recommendations such as requiring the use of face masks.

**Population Health Research Capsule**

What do we already know about this issue?

*COVID-19 led to a reduction in ED visits and had disproportionate health effects among minorities; its impact on ED visits for that population is understudied*.

What was the research question?

How did COVID-19 and safer-at-home orders impact ED utilization and patient-mix at a large, safety-net ED?

What was the major finding of the study?

*Safer-at-home orders were associated with a large, sustained drop in ED use across nearly all patient groups*.

How does this improve population health?

*Identifying patients with disproportionate decreases in ED utilization allows us to prioritize outreach to encourage continued use of necessary healthcare services*.

LAC+USC Medical Center is the largest, county-run hospital in LAC. Annual ED visits average over 150,000 making it one of the busiest EDs in the nation. LAC+USC is situated in a relatively low-income and disproportionately Latino neighborhood, directly adjacent to downtown Los Angeles’s Skid Row; it also provides care for persons detained at the largest jail in the US, the Twin Towers Correctional Facility.^17^,^18^

All data and measures come from the electronic health records (EHR) of patients during these visits; the same EHR system was in place for the entirety of our observation windows. We selected outcomes and measures whose reporting is unchanged over the three-year period. The University of Southern California Institutional Review Board approved all study procedures.

### Outcomes and Measures

To estimate the impact of the COVID-19 pandemic and attendant safer-at-home declarations, the key outcome of interest was weekly ED volume measured as the number of ED encounters, including transfers to other facilities. Weeks were measured from Sunday to Saturday. We report the proportion of all ED visits that are accounted for by various patient, diagnosis, and other measures to describe how ED volume has changed across and within years. In addition to the main outcome of ED volume, we also report the rate at which patients were admitted to inpatient units of the same hospital.

Key patient-level characteristics were collected from patients during ED registration. These characteristics include the following: patient age; gender; race or ethnicity; nativity; home address; and primary language spoken by the patient. In addition to patient-reported characteristics we included measures recorded in the EHR: primary payor for the ED encounter; mode of arrival to the ED (eg, arrival by ambulance); Emergency Severity Index (ESI) triage category; ED disposition; and primary diagnosis at discharge. These patient and encounter-level characteristics are presented as the count or proportion of all weekly ED visits accounted for by these categories.

To present a comprehensive, meaningful estimate of the diagnosis-mix of patients presenting to the ED, we categorized all ED encounters by the primary or first-listed diagnosis code in the patient’s EHR. Those individual *International Classification of Diseases, 10^th^ Rev* diagnosis codes are bundled into the 18 multilevel Clinical Classification Software (CCS) diagnostic categories developed by the Healthcare Cost and Utilization Project (HCUP).^19^ These categories group diagnosis codes by body system (eg, diseases of the circulatory system). We report the 10 most common of these categories observed in our population. In addition, we report a Clinical Classifications Software, Revised (CCSR) diagnosis-based definition of encounters that may be related to COVID-19-specific complaints. COVID-associated respiratory diagnoses includes diagnoses of pneumonia (RSP002); influenza (RSP003); acute bronchitis (RSP005); other specified and unspecified upper respiratory infections and disease (RSP006–7); chronic obstructive pulmonary disease and bronchiectasis (RSP008); asthma (RSP009); pleurisy, pleural effusion, pulmonary collapse (RSP011); respiratory failure, respiratory insufficiency, and respiratory arrest (RSP012); lung disease due to external agents (RSP013); and other specified and unspecified lower respiratory disease (RSP016). All diagnosis categories come from HCUP’s CCSR scheme. The specific breakdown of these diagnoses is available in Appendix Table 1.2.

### Statistical Analysis

This investigation presents descriptive statistics comparing changes in ED volume across years and by week within years. In most cases, we present unadjusted counts or shares of ED volume attributed to encounter-specific characteristics. Where appropriate, tests of difference were performed using Student’s t-test or f-test. To explore potential changes in the geospatial catchment area of LAC+USC during the study period, we examined and mapped the home addresses of patients presenting to the ED by ZIP code. For this study, we considered only visits by patients whose home addresses were in mainland LAC, and excluded visits from patients living on islands or outside LAC, as well as those representing group facilities. Shapefiles of ZIP codes in LAC were obtained from LAC eGIS. To facilitate comparison between periods, the count of visits per ZIP code was rescaled and centered within each nine-week period. Further details on the geographic analyses are included in Appendix 2.

## RESULTS

### Overall Impact on ED Utilization

The LAC+USC Medical Center ED had approximately 56,000 patient encounters in the first 18 weeks of each year for the initial two years of the study period; in 2020, that number dropped by almost 20% to 45,448. These declines in ED utilization were observed broadly by patient characteristics, encounter acuity, and patient diagnoses. Notably, as shown in Table 1, nearly all these decreases came in the second half of our observation period (weeks 10–18) in 2020 with total ED encounters dropping 36% from 27,778 in weeks 1–9 to 17,670 in weeks 10–18. Across all outcome measures and patient or encounter characteristics recorded in our study, total ED volume as measured in counts decreased from the first half of the observation period to the second half of the observation period in 2020 (Table 1 and Appendix Table 1.1; *P*<0.05 in all but five comparisons). The magnitude of these reductions ranged from a 16% decrease to a 58% decrease with an average decrease of about 33% (authors’ calculations based on Table 1).

The geographic distribution of those reductions was uniform. Of all ED visits where patients reported a home ZIP code within LAC, comparing visits in the first half of the 2020 observation window to the second, there was no change in the relative density of ED visits by ZIP code (Appendix Figure 2.1). As a result, the service area of LAC+USC remained uniform as patients were similarly likely to visit the hospital across ZIP codes. More detail on the geographic distribution of visits is available in Appendix 2.

**Figure 1:**
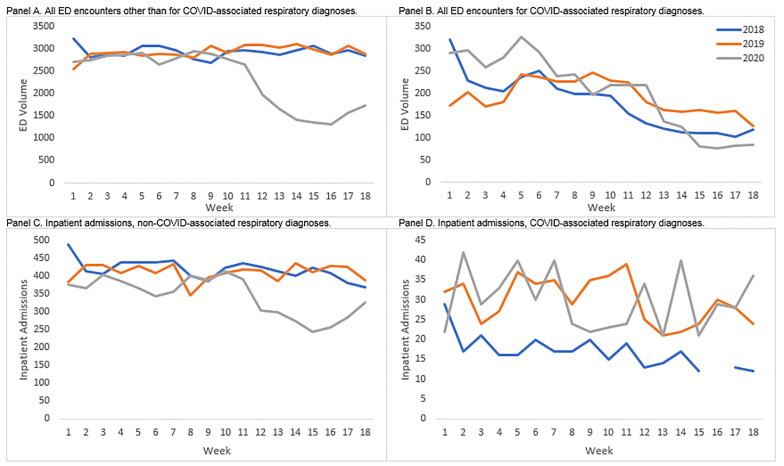
Total emergency department volume and inpatient admissions by week, year, and diagnosis. Notes: Values representing fewer than 10 encounters are omitted. COVID-associated respiratory diagnoses include pneumonia, influenza, acute bronchitis, other specified and unspecified upper respiratory infections and disease, chronic obstructive pulmonary disease and bronchiectasis, asthma, pleurisy, pleural effusion, pulmonary collapse, respiratory failure, respiratory insufficiency, respiratory arrest, lung disease due to external agents, and other specified and unspecified lower respiratory disease. *COVID*, corona virus 2019; *ED*, emergency department.

These decreases also coincided with the safer-at-home declarations issued on March 19, 2020 (week 12 of our observation period in 2020). As shown in Figure 1, Panel A, the reduction in ED encounters occurred quickly and sharply in weeks 12–16, just after the announcement of the safer-at-home declaration. At its lowest point, ED encounters were 50% lower in week 16 compared to week 11, just before the order was issued. A similar pattern emerged for inpatient admissions shown in Figure 1, Panel C, with a sharp decrease after the declaration, although a less dramatic drop-off in the number of admissions; the lowest level of admissions occurred in week 15, representing a 38% reduction relative to week 11, before the order was issued.

### Impact on ED Utilization for Respiratory Diagnoses

The only patient group that saw an increase in the number of ED encounters in the first 18 weeks of 2020 as compared to the same weeks in 2018 or 2019 were patients whose primary diagnosis was categorized as a COVID-associated respiratory diagnosis or a disease of the respiratory system. Depending on the definition and comparison year used, those increases ranged from 444 (12% increase) to 542 (10% increase) visits over the 18-week period (Appendix Tables 1.1 and 1.2). Despite that overall increase in total visits by patients with respiratory diagnoses in 2020, the number of COVID-associated respiratory cases in the ED declined across the weeks with a sharp downturn in the week immediately following the safer-at-home declaration (a 38% reduction relative to week 11).

As shown in Figure 1, Panel B, these cases were elevated relative to prior years in weeks 1–8 (20–34% higher, depending on the comparison year), but after their sharp downturn in week 12, they leveled off at a lower level than in the two prior years by week 15 (26% to 46% lower for weeks 15–18 depending on the comparison year). While much of this reduction came in the form of fewer ED encounters or inpatient admissions for patients with relatively mild complaints, such as asthma, influenza, and other upper respiratory complaints, the within-diagnosis admission rate of patients with COVID-associated respiratory diagnoses nearly doubled from the first to the second half of our 2020 observation period from 11.7% to 20.6% (Appendix Table 1.2). As shown in Figure 1, Panel D, admissions for COVID-associated respiratory diagnoses climbed—particularly from weeks 15 to 18—where they went from about 20 per week to about 35 per week (Figure 1, Panel D). These admissions were concentrated in patients diagnosed with the more serious diagnoses of pneumonia and respiratory failure, insufficiency, or arrest (Appendix Table 1.2).

### What Patients Remain at the ED?

**Figure 2:**
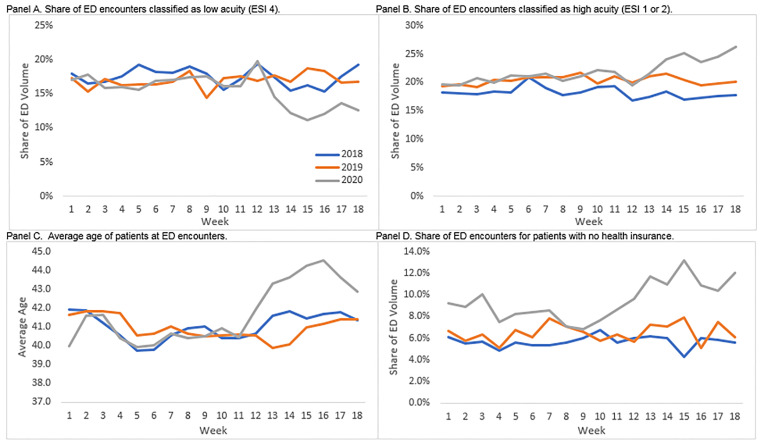
Distribution of emergency department (ED) volume by week, year, and selected characteristics.

While there was an across-the-board decrease in ED utilization, certain groups saw *relative* increases in their share of ED volume as they continued to seek care in the ED more frequently than other patient groups. Comparing weeks 1–9 to weeks 10–18 in 2020 shows that the rate of inpatient admissions grew by about four percentage points and the rate of intensive care unit admissions increased by just under one percentage point (both differences *P*<0.001). The share of ED encounters classified as high acuity (ESI 1 and 2) grew by more than two percentage points while the share classified as relatively low acuity (ESI 4) dropped by a similar amount. There was a similar difference between the early and late periods for patients arriving by ambulance. Interestingly, this shift in the distribution of ED encounters from lower to higher acuity occurred just after the introduction of the safer-at-home regulations in week 12 (Figure 2, Panels A and B). There was no similar trend in prior years.

In addition to higher acuity patients accounting for a disproportionate share of ED utilization relative to prior years and to the first half of our observation periods, the patients who continued to visit the ED tended to be older, more likely to be male, Black, uninsured or using non-Medicaid, publicly-provided insurance programs, or English speakers. Just as the shifts in the distribution of encounters by acuity were observed just after the safer-at-home declarations, so too were the increases in the average age of the patients, which rose by between 2–3 years from week 11 to weeks 13–16 (Figure 2, Panel C). The shift in the distribution of ED encounters for uninsured patients came slightly before the safer-at-home declarations and appears to level off at a higher relative share in weeks 12–13 (Figure 2, Panel D).

**Figure 3:**
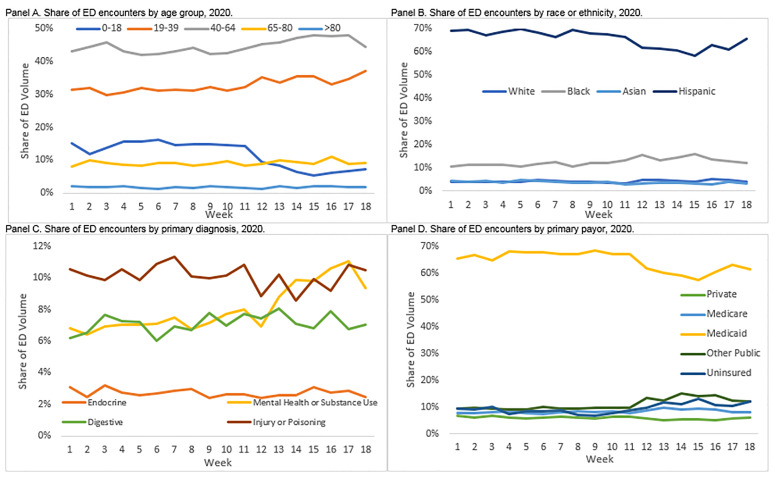
Distribution of emergency department (ED) volume by week and selected characteristics, 2020.

Looking by week in 2020, one of the more notable shifts in the ED distribution was the strong, persistent reduction in the share of encounters for children. As shown in Figure 3, Panel A, there were slight, relative increases in the share of patients aged 19–64 after week 11, but children went from about 14% of all ED visits in week 11 to just 5% in week 15. Another notable shift was in the distribution of diagnoses of patients (Figure 3, Panel C). Trauma diagnoses (injury or poisoning), digestive diagnoses, and endocrine diagnoses saw relatively little fluctuation in their share of ED volume across weeks in 2020. In contrast, the share of ED volume accounted for by patients with mental health and substance use diagnoses increased dramatically just after the safer-at-home declarations in week 12. These patients saw a relative increase in their share of ED volume of about four percentage points (from about 7% to 11%).

### What Patients are Returning to the ED?

By the last four weeks of our observation period in 2020, some patient groups had begun returning to the ED for acute care. Among all non-respiratory encounters, the sharp reductions in ED volume leveled off in week 15 and began to change direction in weeks 17 and 18 (Figure 1, Panel A); increases in inpatient admissions started as soon as week 15 (Figure 1, Panel C). As noted above, one of the main drivers of this increase appears to be an increase in the number of patients coming to the ED for mental health and substance use diagnoses. In addition, beginning in week 15, the observed drop-off in the share of ED encounters for Hispanic patients or patients paying with Medicaid was reduced but not eliminated (Figure 3, Panels B and D). As these are two populations that account for a large proportion of LAC+USC volume historically, their return contributes to the broader reversal in trend. Despite these initial returns of selected patient populations, there were still more than 1000 fewer ED encounters in 2020 as compared to 2018 or 2019 in the final week of our observation window, a nearly 40% reduction in volume compared to prior years.

## DISCUSSION

In this analysis of administrative patient data from a large, urban, safety-net ED, we found drastic reductions as well as differential changes in ED utilization based on diagnoses and demographic subgroups. The first half of our observation period shows largely identical trends in ED utilization across time, patient populations, and diagnosis mix. Despite those similar trends, there was a sharp, marked reduction in ED utilization after the implementation of safer-at-home measures in LAC, consistent with existing studies on ED utilization.^12^ The initial reduction was fairly uniform across patient characteristics and diagnoses, and that reduction relative to prior years, although somewhat attenuated, continued for the duration of our 2020 observation period. Notably, even patients with a collection of diagnoses that are likely to be associated with symptoms of COVID-19 saw a strong reduction in ED utilization in the second half of our observation period in 2020. While much of this reduction came in the form of fewer ED encounters or inpatient admissions for patients with relatively mild respiratory complaints, the admission rate of patients with COVID-associated respiratory diagnoses nearly doubled from the first to the second half of our 2020 observation period. This finding is broadly consistent with existing literature showing an increase in inpatient admission rates as the pandemic intensifies in a given area.^12^

However, what existing research has not documented is that while all classes of patients were less likely to seek care in the ED in the wake of the safer-at-home orders and spread of COVID-19, certain groups saw extreme reductions in utilization while others saw relatively mild decreases. Our findings show some initial evidence that as COVID-19 spread, the patients who continued to visit the ED were relatively sicker, as shown by higher rates of inpatient admissions, higher acuity scores, and higher rates of transport by ambulance. While we cannot rule out the possibility that a relatively empty hospital led to increased admissions – although our clinical experience would combat that explanation – this initial evidence provides an important avenue for future research.

In addition to shifts in patient acuity, we also catalogued shifts in the distribution of ED volume by patient diagnoses and demographics. Most troubling was the sharp increase in the share of patients diagnosed with mental health or substance use disorders. It has been posited that COVID-19 policy responses aimed at curbing disease spread and the resulting economic downturn were likely to have adverse impacts on mental health and substance abuse disorders.^20^,^21^ Yao and colleagues describe how the COVID-19 pandemic triggered a “parallel epidemic” of fear, anxiety, and depression.^22^ Our findings provide early evidence that this effect may be seen almost immediately.

Our findings are consistent with those from abroad that report increased levels of stress and anxiety concurrent with the COVID-19 pandemic.^23^ At its peak in China, more than half of surveyed respondents rated the psychological impact of COVID-19 as moderate to severe, and about one-third reported moderate to severe anxiety.^24^ It is unclear whether this increase in proportion of visits for mental health and substance abuse disorders was driven primarily by increased anxiety and stress, the economic impact of the pandemic, reduced access to medication, reduced services, or isolation from personal support systems, but all explanations provide fruitful avenues for further research.

Our study also adds clarity to the phenomenon of delaying care observed in the time of COVID. Nationally, nearly half of Americans reported that they or a family member skipped or delayed seeking care.^25^ Over 20% of those respondents believed the medical condition worsened due to the delay.^25^ However, evidence-based estimates of the delay in care for emergency conditions or the timeframe for patients to return are few. There is also a lack of clarity regarding which patients were delaying necessary care: Were those patients at higher risk for severe COVID-19 complications avoiding the ED due to a perceived elevated risk of exposure to germs, or were patients who perceived themselves as healthy enough to withstand their ailments without hospital care more likely to avoid the ED?

In our population, we found disproportionate decreases among several patient subgroups: pediatric patients; Hispanic patients; and patients with Medicaid insurance. These three subgroups represent relatively healthy groups in our patient population. These patients may perceive that they can safely self-treat symptoms at home or wait to have chronic conditions managed. However, early reports from COVID-19-stricken countries indicate that as pediatric ED visits have sharply declined, there are dangerous consequences from lack of access to hospital care.^10^,^26^

In the context of a sanctuary hospital, we must also recognize the chilling effect that a government directive can have on care-seeking behavior; patients may delay necessary emergency care due to fear of the legal ramifications of being found in violation of federal immigration law.^27^ Prior experience has shown these decreases to be small and short term.^28^–^30^ This prolonged decrease, which was only partially rebounding at the end of our study period, does not follow prior patterns with fear of legal ramifications.

## LIMITATIONS

This study has several limitations that affect its generalizability. Our data reflect the impact of the COVID-19 pandemic and “safer-at-home” declaration on a single hospital within a major metropolitan area. It is possible that reductions in ED visits at LAC+USC Medical Center were offset by utilization at other area hospitals. However, national studies, review of data from other public facilities, and personal discussions suggest all area hospitals saw a decrease in visits.^10^ Further, there was a collection of institution-specific policy changes made with respect to COVID-19 testing, triage, and admission decisions in the interest of public health and safety (often related to testing availability) during our observation window in 2020 that did not occur in earlier years and may have occurred differently at other hospitals.

In addition, we employed primary diagnosis, rather than chief complaint, because it is more reliably coded in our underlying data. We do note that primary diagnosis was missing for a disproportionate share of ED encounters in 2020 as compared to 2019 and 2018. One explanation is that diagnosis codes are added to EHRs after the fact and not all charts may have been processed prior to our analysis. The other explanation is that most missing diagnoses were for encounters where patients left without being seen by a physician or left before treatment was complete (99% in 2018, 75% in 2019 and the first nine weeks of 2020, and 54% for the second nine weeks of 2020). While this could have skewed our findings, we could not assign diagnoses or likely diagnoses without more information. In addition, we only captured primary diagnosis rather than all diagnoses because of the inconsistent coding of non-primary diagnoses in the underlying data; this decision may have led us to miss cases of interest where the diagnosis was captured in a non-primary diagnosis variable.

Finally, we caution that the findings in our study represent descriptive rather than causal relationships between the spread of COVID-19, implementation of safer-at-home declarations, and ED utilization. Future studies should work to establish whether the descriptive relationship we observed is causal and whether fear of contracting COVID-19, breaking safer-at-home declarations, or other factors are the primary mechanism explaining the observed patterns in the data.

## CONCLUSION

### Public Health Implications

Our findings point to an abrupt, discontinuous impact of COVID-19 on ED utilization with a slow return as safer-at-home orders weakened in Los Angeles County. Despite this turnaround, there were still 40% fewer ED visits in the final week of our observation period compared to prior years. What remains to be determined is what the medium- and long-term impact of this strong reduction in utilization means for patient outcomes, disease control, and the health of the community.

## Supplementary Information



## Figures and Tables

**Table 1:** Description of emergency department patients and utilization, 2018, 2019, and 2020.

	2018 Weeks 1–9		2019 Weeks 1–9		2020 Weeks 1–9		P-value	2018 Weeks 10–18		2019 Weeks 10–18		2020 Weeks 10–18		P-value

Outcome Measures	N	%	N	%	N	%		N	%	N	%	N	%	
Total ED Volume	28,436	(100.0)	27,678	(100.0)	27,778	(100.0)		27,594	(100.0)	28,598	(100.0)	17,670	(100.0)	
Inpatient Admissions	4,025	(14.2)	3,953	(14.3)	3,668	(13.2)	<0.001	3,806	(13.8)	3,968	(13.9)	3,045	(17.2)	<0.001
ICU Admissions	876	(3.1)	966	(3.5)	902	(3.3)	0.023	834	(3.0)	948	(3.3)	750	(4.2)	<0.001
Patient Characteristics														
0–18 y.o.	4,187	(14.7)	3,726	(13.5)	4,087	(14.7)	<0.001	3,585	(13.0)	3,956	(13.8)	1,696	(9.6)	<0.001
19–39 y.o.	9,012	(31.7)	8,853	(32.0)	8,708	(31.4)	0.272	8,960	(32.5)	9,221	(32.2)	5,997	(33.9)	<0.001
40–64 y.o.	12,087	(42.5)	12,101	(43.7)	12,039	(43.3)	0.012	12,124	(43.9)	12,489	(43.7)	8,021	(45.4)	0.001
65–80 y.o.	2,573	(9.1)	2,506	(9.1)	2,440	(8.8)	0.443	2,411	(8.7)	2,504	(8.8)	1,644	(9.3)	0.076
>80 y.o	574	(2.0)	492	(1.8)	498	(1.8)	0.060	509	(1.8)	427	(1.5)	305	(1.7)	0.005
Male	16,081	(56.6)	15,731	(56.8)	15,404	(55.5)	0.002	15,772	(57.2)	15,930	(55.7)	10,691	(60.5)	<0.001
Patient Acuity^+^														
ESI 1	216	(0.8)	287	(1.0)	303	(1.1)	<0.001	198	(0.7)	268	(0.9)	235	(1.3)	<0.001
ESI 2	5,059	(17.8)	5,360	(19.4)	5,409	(19.5)	<0.001	4,745	(17.2)	5,564	(19.5)	3,810	(21.6)	<0.001
ESI 3	15,643	(55.0)	15,197	(54.9)	15,256	(54.9)	0.964	15,475	(56.1)	15,494	(54.2)	9,232	(52.3)	<0.001
ESI 4	5,106	(18.0)	4,560	(16.5)	4,674	(16.8)	<0.001	4,702	(17.0)	4,984	(17.4)	2,611	(14.8)	<0.001
ESI 5	711	(2.5)	613	(2.2)	654	(2.4)	0.083	741	(2.7)	590	(2.1)	352	(2.0)	<0.001
Ambulance^$^	5,524	(19.4)	5,035	(18.2)	4,518	(16.3)	<0.001	5,610	(20.3)	5,129	(17.9)	3,483	(19.7)	<0.001
Patient Language														
English	15,219	(53.5)	14,791	(53.4)	14,553	(52.4)	<0.001	14,959	(54.2)	15,109	(52.8)	10,081	(57.1)	<0.001
Spanish	11,833	(41.6)	11,966	(43.2)	12,251	(44.1)	<0.001	11,402	(41.3)	12,493	(43.7)	7,022	(39.7)	<0.001
Other	1,213	(4.3)	778	(2.8)	799	(2.9)	<0.001	1,097	(4.0)	764	(2.7)	486	(2.8)	<0.001
Patient Nativity														
US-born	15,019	(53.1)	13,961	(50.7)	13,816	(50.1)	<0.001	14,372	(52.3)	14,439	(50.9)	8,972	(51.0)	0.001
Undocumented^*^	171	(0.6)	143	(0.5)	175	(0.6)	0.190	136	(0.5)	232	(0.8)	81	(0.5)	<0.001

	2018 Weeks 1–9		2019 Weeks 1–9		2020 Weeks 1–9		P-value	2018 Weeks 10–18		2019 Weeks 10–18		2020 Weeks 10–18		P-value

Patient Primary Payor	N	%	N	%	N	%		N	%	N	%	N	%	
Private Insurance	1,150	(4.0)	1,042	(3.8)	1,722	(6.2)	<0.001	1,103	(4.0)	1,891	(6.6)	1,040	(5.9)	<0.001
Medicaid	20,508	(72.1)	19,473	(70.4)	18,592	(66.9)	<0.001	19,509	(70.7)	19,412	(67.9)	11,069	(62.6)	<0.001
Medicare	2,328	(8.2)	2,232	(8.1)	2,234	(8.0)	0.795	2,249	(8.2)	2,248	(7.9)	1,526	(8.6)	0.012
Other Public	2,572	(9.0)	2,796	(10.1)	2,624	(9.5)	<0.001	2,836	(10.3)	2,848	(10.0)	2,147	(12.2)	<0.001
Uninsured	1,583	(5.6)	1,791	(6.5)	2,308	(8.3)	<0.001	1,597	(5.8)	1,871	(6.5)	1,797	(10.2)	<0.001

Patient Race or Ethnicity														
White	1,078	(3.8)	1,028	(3.7)	1,150	(4.1)	0.022	1,100	(4.0)	1,007	(3.5)	745	(4.2)	<0.001
Black	3,620	(12.7)	3,195	(11.5)	3,149	(11.3)	<0.001	3,487	(12.6)	3,389	(11.9)	2,398	(13.6)	<0.001
Asian	1,169	(4.1)	1,100	(4.0)	1,121	(4.0)	0.712	1,105	(4.0)	1,073	(3.8)	607	(3.4)	0.008
Hispanic	19,061	(67.0)	18,887	(68.2)	19,025	(68.5)	<0.001	18,367	(66.6)	19,624	(68.6)	11,214	(63.5)	<0.001
Unknown	3,264	(11.5)	3,251	(11.8)	3,098	(11.2)	0.090	3,318	(12.0)	3,223	(11.3)	2,577	(14.6)	<0.001

*ED*, emergency department; *ICU*, intensive care unit; *y.o.*, years old; *ESI*, Emergency Severity Index.
