# Supplementary material for: Who Stayed Home Under Safer-at-Home? Impacts of COVID-19 on Volume and Patient-Mix at an Emergency Department
Source: West J Emerg Med. 2021 Feb 8;22(2):234–43. doi: 10.5811/westjem.2020.12.49234 (PMC7972372; doi:10.5811/westjem.2020.12.49234)
Supplement: Supplementary file 1 [file wjem-22-234-s001.docx]

**Appendix 1. Supplementary Tables**

Appendix Table 1.1 Description of ED Volume and Admissions by Diagnosis, 2018, 2019, and 2020

|  | **2018** | | **2019** | | **2020** | | **p-value** | **2018** | | **2019** | | **2020** | | **p-value** |
| --- | --- | --- | --- | --- | --- | --- | --- | --- | --- | --- | --- | --- | --- | --- |
|  | Week 1-9 | | Week 1-9 | | Week 1-9 | |  | Week 10-18 | | Week 10-18 | | Week 10-18 | |  |
| **Total ED Volume** | N | % | N | % | N | % |  | N | % | N | % | N | % |  |
| COVID-Associated Respiratory Diagnosis | 2,062 | 7.3 | 1,904 | 6.9 | 2,421 | 8.7 | <0.001 | 1,156 | 4.2 | 1,559 | 5.5 | 1,241 | 7.0 | <0.001 |
| Endocrine, nutritional, and metabolic diseases and immunity disorders | 1,373 | 4.8 | 1,215 | 4.4 | 773 | 2.8 | <0.001 | 1,375 | 5.0 | 1,147 | 4.0 | 466 | 2.6 | <0.001 |
| Mental health or substance use | 2,710 | 9.5 | 2,362 | 8.5 | 1,939 | 7.0 | <0.001 | 2,841 | 10.3 | 2,471 | 8.6 | 1,565 | 8.9 | <0.001 |
| Diseases of the nervous system and sense organs | 2,546 | 9.0 | 2,473 | 8.9 | 2,394 | 8.6 | 0.293 | 2,567 | 9.3 | 2,578 | 9.0 | 1,277 | 7.2 | <0.001 |
| Diseases of the circulatory system | 2,105 | 7.4 | 2,412 | 8.7 | 2,055 | 7.4 | <0.001 | 2,217 | 8.0 | 2,391 | 8.4 | 1,441 | 8.2 | 0.363 |
| Diseases of the respiratory system | 2,955 | 10.4 | 2,824 | 10.2 | 3,283 | 11.8 | <0.001 | 1,873 | 6.8 | 2,403 | 8.4 | 2,087 | 11.8 | <0.001 |
| Diseases of the digestive system | 1,812 | 6.4 | 1,995 | 7.2 | 1,927 | 6.9 | <0.001 | 1,872 | 6.8 | 2,039 | 7.1 | 1,294 | 7.3 | 0.072 |
| Diseases of the genitourinary system | 1,503 | 5.3 | 1,604 | 5.8 | 1,621 | 5.8 | 0.007 | 1,526 | 5.5 | 1,642 | 5.7 | 1,027 | 5.8 | 0.380 |
| Diseases of the musculoskeletal system and connective tissue | 1,887 | 6.6 | 1,936 | 7.0 | 1,983 | 7.1 | 0.053 | 1,896 | 6.9 | 2,122 | 7.4 | 1,076 | 6.1 | <0.001 |
| Injury and poisoning | 2,050 | 7.2 | 2,136 | 7.7 | 2,879 | 10.4 | <0.001 | 2,079 | 7.5 | 2,625 | 9.2 | 1,764 | 10.0 | <0.001 |
| Symptoms; signs; and ill-defined conditions and factors influencing health status | 3,312 | 11.7 | 2,914 | 10.5 | 2,488 | 9.0 | <0.001 | 3,331 | 12.1 | 3,003 | 10.5 | 1,681 | 9.5 | <0.001 |
| Missing diagnoses | 425 | 1.5 | 929 | 3.4 | 2,981 | 10.7 | <0.001 | 385 | 1.4 | 1,459 | 5.1 | 1,449 | 8.2 | <0.001 |
| **Total Inpatient Admissions and Admission Rate** |  |  |  |  |  |  |  |  |  |  |  |  |  |  |
| COVID-Associated Respiratory Diagnosis | 173 | 8.4 | 287 | 15.1 | 282 | 11.7 | <0.001 | 122 | 10.6 | 249 | 16.0 | 256 | 20.6 | <0.001 |
| Endocrine, nutritional, and metabolic diseases and immunity disorders | 485 | 35.3 | 349 | 28.7 | 310 | 40.1 | <0.001 | 458 | 33.3 | 347 | 30.3 | 203 | 43.6 | <0.001 |
| Mental health or substance use | 407 | 15.0 | 301 | 12.7 | 187 | 9.6 | <0.001 | 401 | 14.1 | 285 | 11.5 | 134 | 8.6 | <0.001 |
| Diseases of the nervous system and sense organs | 206 | 8.1 | 201 | 8.1 | 153 | 6.4 | 0.032 | 224 | 8.7 | 208 | 8.1 | 141 | 11.0 | 0.009 |
| Diseases of the circulatory system | 376 | 17.9 | 679 | 28.2 | 692 | 33.7 | <0.001 | 374 | 16.9 | 644 | 26.9 | 579 | 40.2 | <0.001 |
| Diseases of the respiratory system | 264 | 8.9 | 449 | 15.9 | 428 | 13.0 | <0.001 | 203 | 10.8 | 416 | 17.3 | 474 | 22.7 | <0.001 |
| Diseases of the digestive system | 299 | 16.5 | 417 | 20.9 | 417 | 21.6 | <0.001 | 316 | 16.9 | 426 | 20.9 | 275 | 21.3 | 0.001 |
| Diseases of the genitourinary system | 189 | 12.6 | 208 | 13.0 | 213 | 13.1 | 0.891 | 159 | 10.4 | 218 | 13.3 | 163 | 15.9 | <0.001 |
| Diseases of the musculoskeletal system and connective tissue | 121 | 6.4 | 152 | 7.9 | 148 | 7.5 | 0.208 | 102 | 5.4 | 140 | 6.6 | 97 | 9.0 | 0.001 |
| Injury and poisoning | 263 | 12.8 | 315 | 14.8 | 298 | 10.4 | <0.001 | 256 | 12.3 | 353 | 13.5 | 267 | 15.1 | 0.038 |
| Symptoms; signs; and ill-defined conditions and factors influencing health status | 416 | 12.6 | 198 | 6.8 | 237 | 9.5 | <0.001 | 399 | 12.0 | 241 | 8.0 | 198 | 11.8 | <0.001 |
| Missing diagnoses | -- | -- | 14 | 1.5 | 31 | 1.0 | 0.104 | -- | -- | 14 | 1.0 | 20 | 1.4 | 0.142 |

Notes: -- indicates fewer than 10 observations

COVID-Associated Respiratory Diagnoses include: pneumonia, influenza, acute bronchitis, other specified and unspecified upper respiratory infections and disease, COPD and bronchiectasis, asthma, pleurisy, pleural effusion, pulmonary collapse, respiratory failure, respiratory insufficiency, respiratory arrest, lung disease due to external agents, and other specified and unspecified lower respiratory disease. Detailed breakdowns are in Appendix Table 1.2

Appendix Table 1.2 Distribution of ED Volume, Inpatient Admissions, and Admission Rate by Respiratory or COVID-Associated Diagnosis, 2018, 2019, and 2020

|  | 2018 | | 2019 | | 2020 | |  | 2018 | | 2019 | | 2020 | |
| --- | --- | --- | --- | --- | --- | --- | --- | --- | --- | --- | --- | --- | --- |
|  | Week 1-9 | | Week 1-9 | | Week 1-9 | |  | Week 10-18 | | Week 10-18 | | Week 10-18 | |
|  | **N** | **%** | **N** | **%** | **N** | **%** |  | **N** | **%** | **N** | % | **N** | **%** |
| **Total ED Volume** | 28,436 | (100.0) | 27,678 | (100.0) | 27,778 | (100.0) |  | 27,594 | (100.0) | 28,598 | (100.0) | 17,670 | (100.0) |
| Diseases of the respiratory system | 2955 | (10.4) | 2824 | (10.2) | 3283 | (11.8) |  | 1873 | (6.8) | 2403 | (8.4) | 2087 | (11.8) |
| COVID-Associated Respiratory Diagnosis | 2062 | (7.3) | 1904 | (6.9) | 2421 | (8.7) |  | 1156 | (4.2) | 1559 | (5.5) | 1241 | (7.0) |
| Pneumonia (except caused by tuberculosis) | 120 | (0.4) | 171 | (0.6) | 228 | (0.8) |  | 84 | (0.3) | 169 | (0.6) | 186 | (1.1) |
| Influenza | 423 | (1.5) | 347 | (1.3) | 630 | (2.3) |  | 77 | (0.3) | 164 | (0.6) | 59 | (0.3) |
| Acute bronchitis | 115 | (0.4) | 98 | (0.4) | 111 | (0.4) |  | 50 | (0.2) | 58 | (0.2) | 35 | (0.2) |
| Other specified upper respiratory infections | 904 | (3.2) | 789 | (2.9) | 1026 | (3.7) |  | 552 | (2.0) | 674 | (2.4) | 605 | (3.4) |
| Other specified and unspecified upper respiratory disease | 78 | (0.3) | 50 | (0.2) | 43 | (0.2) |  | 40 | (0.1) | 53 | (0.2) | 31 | (0.2) |
| COPD and bronchiestasis | 61 | (0.2) | 86 | (0.3) | 69 | (0.3) |  | 60 | (0.2) | 65 | (0.2) | 59 | (0.3) |
| Asthma | 279 | (1.0) | 220 | (0.8) | 168 | (0.6) |  | 210 | (0.8) | 247 | (0.9) | 113 | (0.6) |
| Pleurisy, pleural effusion and pulmonary collapse | 23 | (0.1) | 34 | (0.1) | 34 | (0.1) |  | 40 | (0.1) | 28 | (0.1) | 26 | (0.2) |
| Respiratory failure, insufficiency, arrest | 32 | (0.1) | 75 | (0.3) | 81 | (0.3) |  | 26 | (0.1) | 70 | (0.2) | 104 | (0.6) |
| Other specified and unspecified lower respiratory disease | 27 | (0.1) | 34 | (0.1) | 30 | (0.1) |  | 16 | (0.1) | 30 | (0.1) | 22 | (0.1) |
|  |  |  |  |  |  |  |  |  |  |  |  |  |  |
| **Total Inpatient Admissions** | 4,025 | (100.0) | 3,953 | (100.0) | 3,668 | (100.0) |  | 3,806 | (100.0) | 3,968 | (100.0) | 3,045 | (100.0) |
| Diseases of the respiratory system | 264 | (6.6) | 449 | (11.4) | 428 | (11.7) |  | 203 | (5.3) | 416 | (10.5) | 474 | (15.6) |
| COVID-Associated Respiratory Diagnosis | 173 | (4.3) | 287 | (7.3) | 282 | (7.7) |  | 122 | (3.2) | 249 | (6.3) | 256 | (8.4) |
| Pneumonia (except caused by tuberculosis) | 34 | (0.8) | 69 | (1.7) | 79 | (2.2) |  | 24 | (0.6) | 64 | (1.6) | 93 | (3.1) |
| COPD and bronchiestasis | 11 | (0.3) | 28 | (0.7) | 14 | (0.4) |  | 11 | (0.3) | 21 | (0.5) | 18 | (0.6) |
| Asthma | 19 | (0.5) | 23 | (0.6) | 12 | (0.3) |  | 16 | (0.4) | 32 | (0.8) | 13 | (0.4) |
| Pleurisy, pleural effusion and pulmonary collapse | 12 | (0.3) | 19 | (0.5) | 24 | (0.7) |  | 23 | (0.6) | 20 | (0.5) | 18 | (0.6) |
| Respiratory failure, insufficiency, arrest | 30 | (0.7) | 71 | (1.8) | 69 | (1.9) |  | 24 | (0.6) | 67 | (1.7) | 98 | (3.2) |
|  |  |  |  |  |  |  |  |  |  |  |  |  |  |
| **Within-Diagnosis Admission Rate** |  |  |  |  |  |  |  |  |  |  |  |  |  |
| Diseases of the respiratory system | 264 | (8.9) | 449 | (15.9) | 428 | (13.0) |  | 203 | (10.8) | 416 | (17.3) | 474 | (22.7) |
| COVID-Associated Respiratory Diagnosis | 173 | (8.4) | 287 | (15.1) | 282 | (11.6) |  | 122 | (10.6) | 249 | (16.0) | 256 | (20.6) |
| Pneumonia (except caused by tuberculosis) | 34 | (28.3) | 69 | (40.4) | 79 | (34.6) |  | 24 | (28.6) | 64 | (37.9) | 93 | (50.0) |
| COPD and bronchiestasis | 11 | (18.0) | 28 | (32.6) | 14 | (20.3) |  | 11 | (18.3) | 21 | (32.3) | 18 | (30.5) |
| Asthma | 19 | (6.8) | 23 | (10.5) | 12 | (7.1) |  | 16 | (7.6) | 32 | (13.0) | 13 | (11.5) |
| Pleurisy, pleural effusion and pulmonary collapse | 12 | (52.2) | 19 | (55.9) | 24 | (70.6) |  | 23 | (57.5) | 20 | (71.4) | 18 | (69.2) |
| Respiratory failure, insufficiency, arrest | 30 | (93.8) | 71 | (94.7) | 69 | (85.2) |  | 24 | (92.3) | 67 | (95.7) | 98 | (94.2) |

Notes: Categories or diagnoses with fewer than 10 observations have been omitted. COVID-Associated Respiratory Diagnosis also includes lung diseases due to external agents. Explicit ICD-10 diagnoses for COVID-19 are excluded because they were not introduced at LAC+USC until the second half of our 2020 observation period.

**Appendix 2: Geospatial Analysis Supplement**

**Methods:** To explore potential changes in the geospatial catchment area of LAC+USC during the study period, we examined and mapped the home address of patients presenting to the ED by zip code. Home addresses were provided by patients on arrival to the ED as part of the normal check-in process and included five-digit zip code. For this study, we considered only visits by patients whose home addresses were in mainland LAC, and excluded visits from patients living on islands or outside LAC. Shapefiles of zip codes in LAC were obtained from LAC eGIS.[1] To remove potential geospatial bias toward group facilities, we manually examined the top 10 most frequent addresses. All 10 of these addresses represent group facilities including jails, homeless shelters, hospitals, or social service offices, and visits from these addresses were excluded from this analysis. Appendix Table 2.1 shows these addresses and their characteristics. Similarly, we excluded visits originating at addresses identified as homeless shelters by the Los Angeles Homeless Service Authority. [2] To facilitate comparison between periods, the count of visits per zip code was rescaled and centered within each nine-week period. All analyses were performed using R, extended by the “tidyverse,” and “tmap” packages. [3-5]

**Results:** During the study period, there were 156,558 visits, of which 141,618 (90.5%) originated from an address within LAC. After screening out group address, a total of 129,272 (82.6%) were available for mapping. Appendix Figure 2.1 shows a choropleth map of the relative density ED visits originating in LAC during 2020, before and after the shelter at home orders. As seen in this figure, there are relatively few changes in the overall pattern of where patients live during the 2020 study period. Appendix Figure 2.2 shows choropleth maps of the relative density of ED visits across the longer study period. As seen in this figure, the overall patterns of where patients live appears to be relatively stable across time.

Appendix Figure 2.1: Relative Density of ED visits in first 18 Weeks of 2020


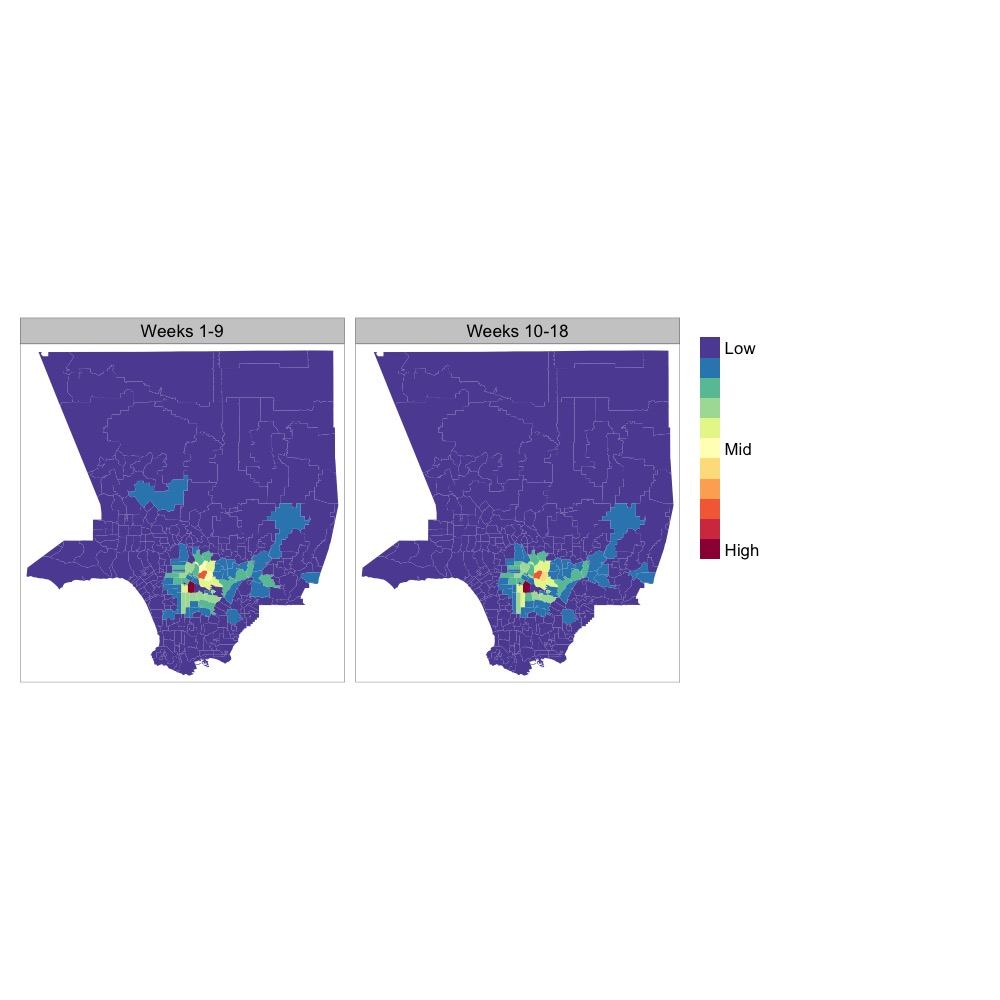


Notes: Choropleth map of the density of ED visits by zip code within LAC during the first 18 weeks of 2020. The scale is relative, with warmer colors representing areas with higher numbers of visits.

**Limitations:** The geospatial component of this study is limited in several ways. First, all addresses are self-reported and not independently verified. It is possible that an individual would give a false address, or that the address would be stated or recorded incorrectly. Collectively, these errors or omissions might alter the resulting catchment areas of the ED, potentially in ways which were not stable across time. Second, the zip code is a relatively large spatial unit which is insensitive to changes that might occur at smaller spatial levels. Further work might geocode patient addresses with higher levels of spatial granularity in order to asses for potential changes at neighborhood levels.

Appendix Table 2.1 – Screened Addresses

|  | Address | Zip | Type | Visits |
| --- | --- | --- | --- | --- |
| 1 | 450 BAUCHET ST | 90012 | Jail | 8256 |
| 2 | 601 S SAN PEDRO ST | 90014 | Homeless Shelter | 460 |
| 3 | 545 S SAN PEDRO ST | 90013 | Homeless Shelter | 441 |
| 4 | 813 E 4TH PL | 90013 | Social Services | 421 |
| 5 | 2415 W 6TH ST | 90057 | Social Services | 356 |
| 6 | 303 E 5TH ST | 90013 | Homeless Shelter | 281 |
| 7 | 1933 S BROADWAY STE 500 | 90007 | Social Services | 243 |
| 8 | 1670 E 120TH ST BLDG 14 I & R | 90059 | Hospital | 241 |
| 9 | 1200 N STATE ST | 90033 | Hospital | 213 |
| 10 | 3804 BROADWAY PL | 90037 | Social Services | 197 |

Notes: Table of the top 10 addresses from which patient visits to the ED originated during the study period. All addresses are in Los Angeles, CA.

**Appendix Figure 2.2: Relative Density of ED visits in first 18 Weeks of 2018, 2019, and 2020**


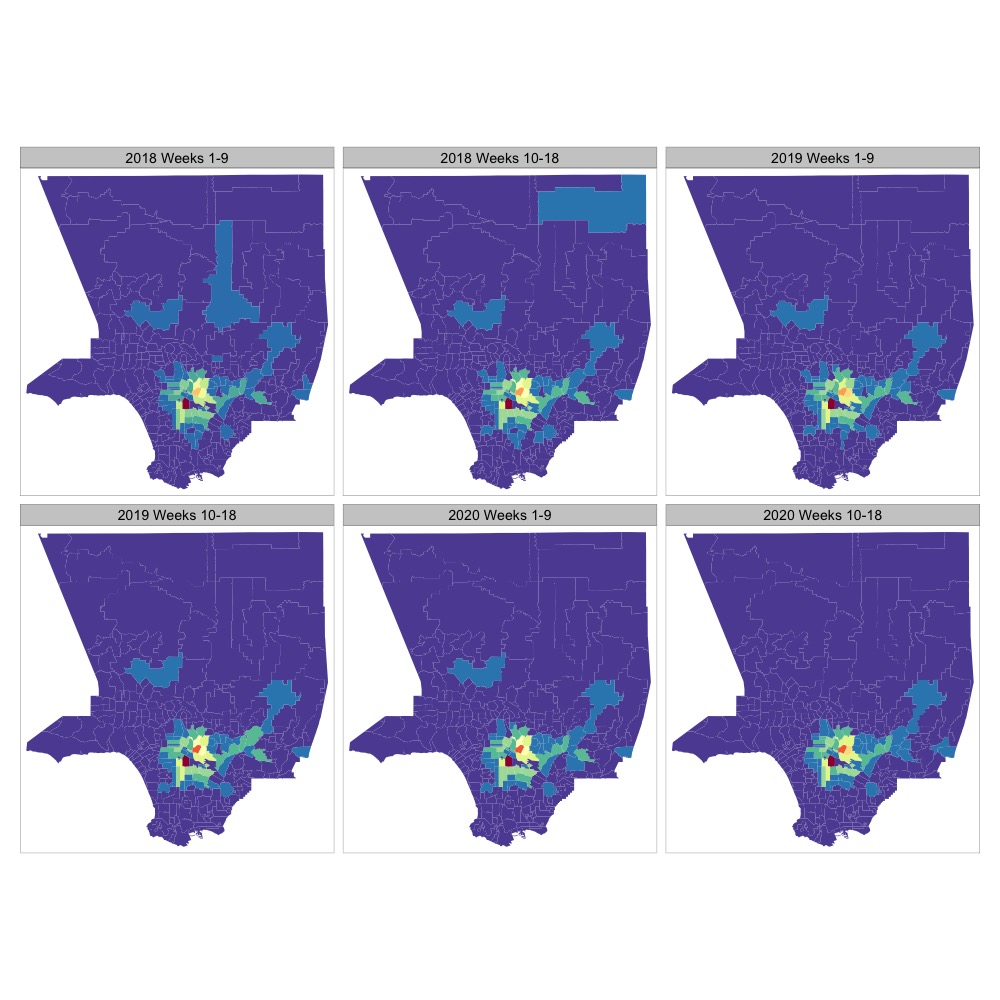


Notes: Choropleth map of the density of ED visits by zip code within LAC during the first 18 weeks of 2018-2020. The scale is relative, with warmer colors representing areas with higher numbers of visits.

**References**

1. County of Los Angeles eGIS. “LA County Zip Code” Available at https://egis-lacounty.hub.arcgis.com/datasets/la-county-zip-code. Accessed 05/2020.
2. Los Angeles Homeless Services Authority. “2019 HOUSING INVENTORY COUNT.” Available at: https://www.lahsa.org/documents?id=3674-2019-housing-inventory-count.xlsx&ref=hc. Accessed: 05/2020.
3. R Core Team. “R: A language and environment for statistical computing.” R Foundation for Statistical Computing, 2018, Vienna, Austria. Available at: www.R-project.org/
4. Tennekes, M. “tmap: Thematic Maps in R.” Journal of Statistical Software, 2018. *84*(6), 1-39.
5. Wickham, H. “tidyverse: Easily Install and Load the 'Tidyverse'.” 2017. Available at: CRAN.R-project.org/package=tidyvers
